# Retraction: Characteristics of cognitive function in patients with cerebellar infarction and its association with lesion location

**DOI:** 10.3389/fnagi.2024.1397905

**Published:** 2024-03-28

**Authors:** Frontiers Editorial Office

*The journal retracts the 04 October 2022 article cited above*.

Following publication, concerns were raised regarding the provenance of the data used in this study. Specifically, during the investigation, which was conducted in accordance with Frontiers' policies, the authors failed to provide appropriate authorization for use of patient data.

Unauthorized data use is a breach of Frontiers' guidelines and those of the Committee on Publication Ethics. As such, this article is being retracted. The authors agree to this retraction.

